# Supplementary material for: Photoinduced multistage phase transitions in Ta2NiSe5
Source: Nat Commun. 2021 Apr 6;12:2050. doi: 10.1038/s41467-021-22345-3 (PMC8024274; doi:10.1038/s41467-021-22345-3)
Supplement: Supplementary file 1 — Supplementary Information [file 41467_2021_22345_MOESM1_ESM.pdf]

Supplementary Information

Photoinduced multistage phase transitions in Ta_2NiSe_5

Q. M. Liu[1‡], D. Wu[1,2‡*], Z. A. Li[3‡], L. Y. Shi[1], Z. X. Wang[1], S. J. Zhang[1], T. Lin[1], T. C. Hu[1], H. F. Tian[3], J.Q. Li[3], T. Dong[1], N. L. Wang[1,4*]

[1]International Center for Quantum Materials, School of Physics, Peking University, Beijing 100871, China

[2]Songshan Lake Materials Laboratory, Dongguan, Guangdong 523808, China

[3]Beijing National Laboratory for Condensed Matter Physics, Institute of Physics, Chinese Academy of Sciences, Beijing 100190, China

[4]Collaborative Innovation Center of Quantum Matter, Beijing, China

I. Device fabrication and temporal spectra of optical excitations

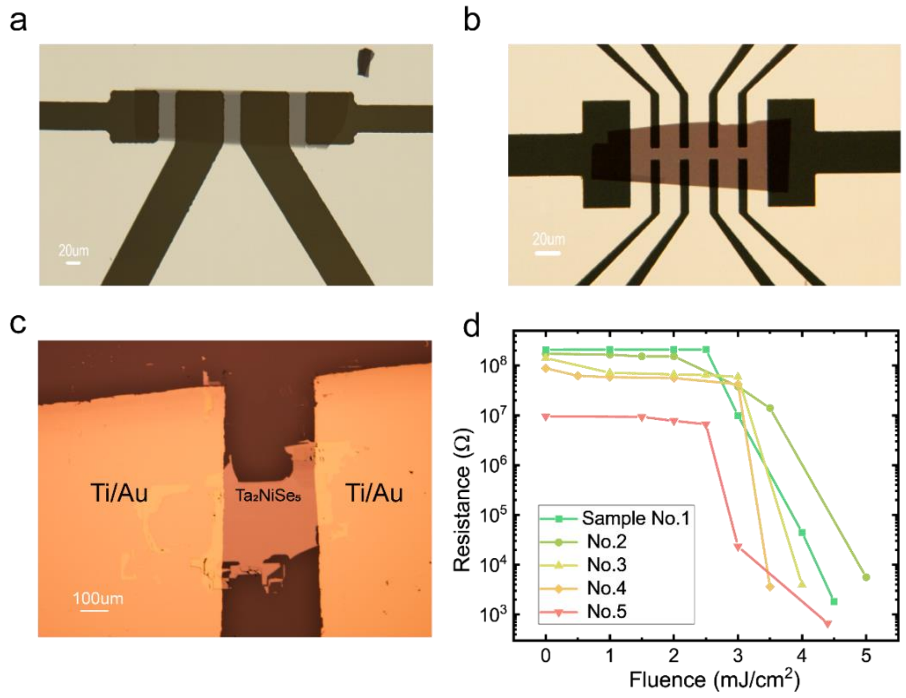

Supplementary Fig. 1. The typical optical images of the Ta_2NiSe_5 devices for experiments of PI resistance switching (a,b) and optical pump-probe (c). The images were taken by the Olympus BX51 Microscope. (d) shows the photoinduced resistance change in different devices.

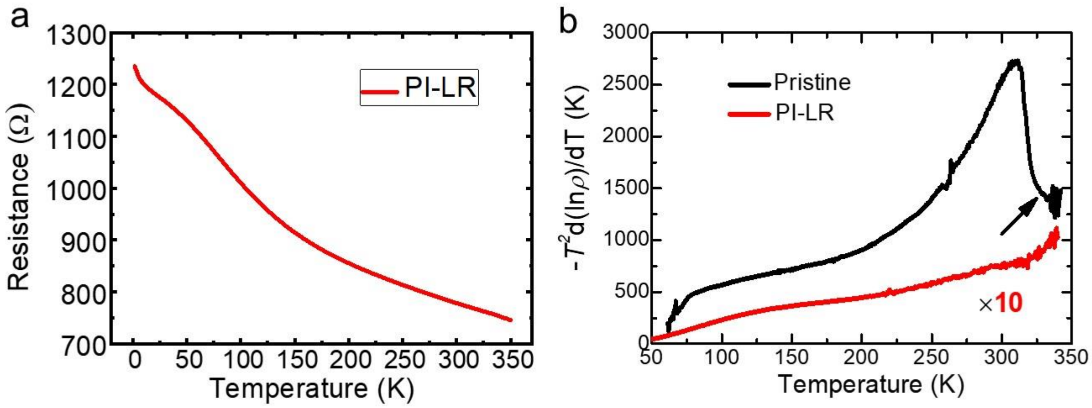

Supplementary Fig. 2. (a)Temperature dependence of the resistance of the PI-LR state of the sample after 30 shots of writing pulse at 3.5 mJ/cm^2. (b) The Arrhenius plot of resistivity for both pristine and PI-LR states. For the pristine sample, the slowly changing region between 100 and 200 K indicates an activation energy of roughly 1000 K (~ 88 meV). The black arrow denotes the anomaly at the plot indicating the ~ 326 K phase transition. While, PI-LR state will yield an extremely small activation energy of ~ 50 K (~ 4 meV), which is about twenty times smaller than the one of pristine sample. There is no detectable anomaly on the PI-LR plot, consistent with that there is only a unique phase for PI-LR state in our measured temperature range.

TEM experiment

Supplementary Fig. 3 shows in-situ heating TEM results of pristine Ta_2NiSe_5 nanoflakes oriented along [110] zone-axis. **Supplementary Fig. 4** shows TEM structural characterization of both pristine and hidden state of Ta_2NiSe_5 nanoflakes oriented along [010] zone-axis. A Schematic Ta lattice shear motion along a-axis in PI-LR state of Ta_2NiSe_5 deduced from TEM experiments is presented in **Supplementary Fig. 5**.

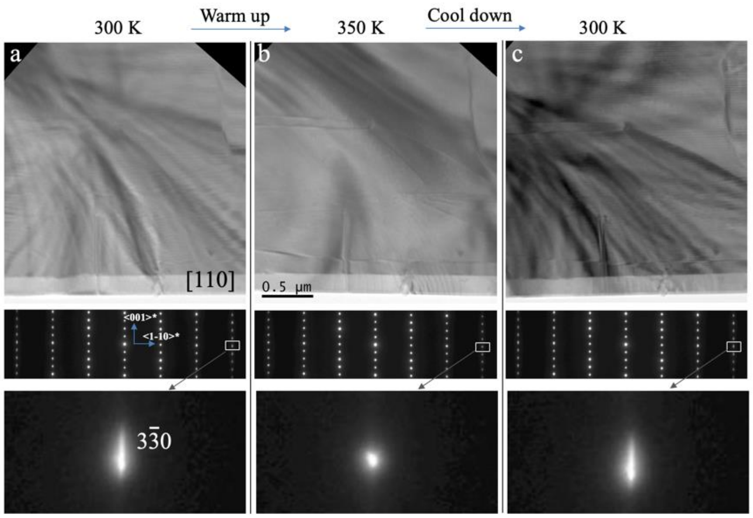

Supplementary Fig. 3. In situ heating TEM experiments of pristine Ta_2NiSe_5 nanoflakes oriented along [110] zone-axis. (a) TEM image, diffraction pattern and enlarged (3-30) reflections taken at room temperature (~ 300 K). TEM image display stripe-like contrast and the corresponding reflections, e.g., (3-30), display splitting or streaking due to the twinning formation in monoclinic Ta_2NiSe_5 phase. (b) Upon heating from 300 K to 350 K, the stripe-like contrast disappears as well as the splitting of (3-30) reflections, indicating that the specimen transforms to orthorhombic phase. (c) Upon cooling from 350 K down to 300 K, the stripe-like contrast appears as well as the splitting of (3-30) reflections, indicating that the specimen transforms to monoclinic phase.

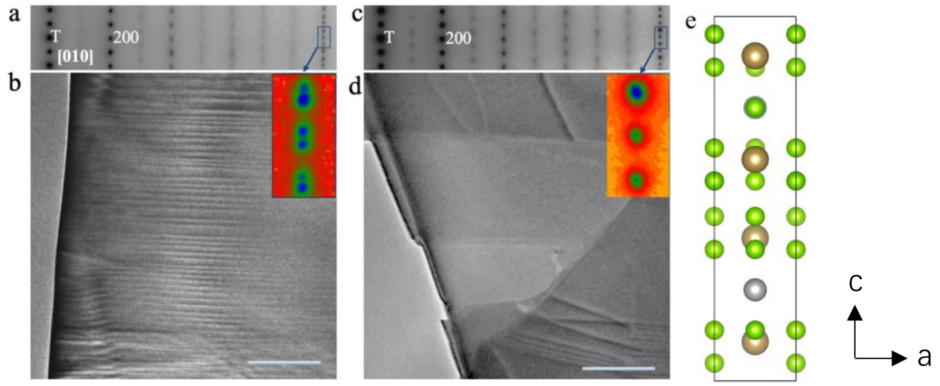

Supplementary Fig. 4. TEM structural characterization of both pristine and hidden state of Ta_2NiSe_5 nanoflakes oriented along [010] zone-axis at room temperature. For pristine Ta_2NiSe_5: (a) [010]-oriented electron diffraction pattern and (b) morphology with stripe-like contrast, and the inset shows the zoom-in part of the splitting of first-order Laue reflections due to the twinning formation. Descriptions for (c-d) are the same as (a-b) but for laser-treated Ta2NiSe5. Inset to (d) shows no splitting of reflections. Letters of T in (a) and (c) mark the transmitted spots. Scale bars in (b) and (d) correspond to 500 nm. False colors scales with the image intensities for the purpose of visual clarity. (e) Projection view along [010] zone-axis of pristine Ta_2NiSe_5.

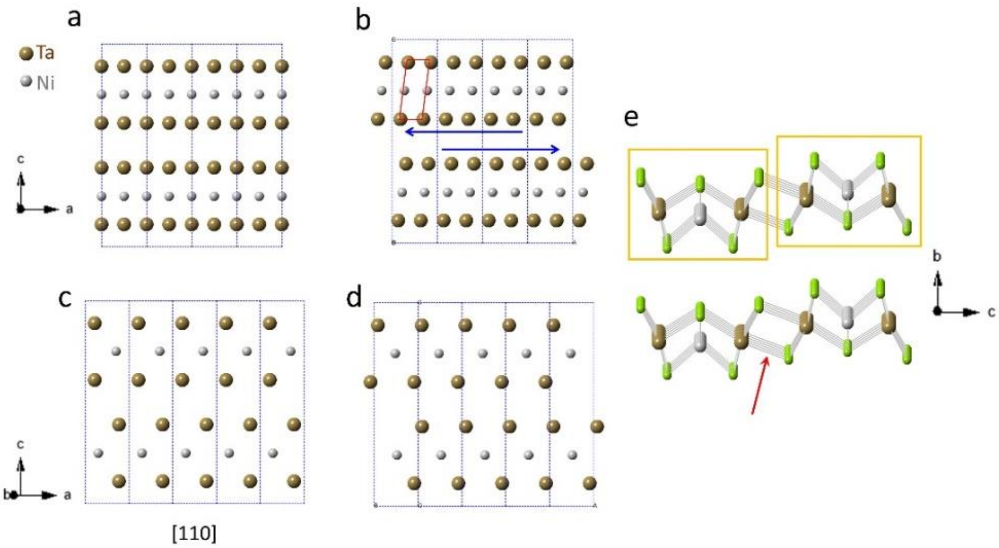

Supplementary Fig. 5. Schematic Ta lattice shear motion along a-axis in PI-LR state of Ta_2NiSe_5 deduced from TEM experiments. (a) Ta- and Ni-chains configuration of pristine orthorhombic state in the view direction of [010]. (b) Same as (a) but in PI-LR state. The red parallelogram denotes the small shear motion of the Ta against the Ni chains. The blue arrows denote the large shear motion occurring between the two Ta-Ni-Ta sub-units along the nearest-neighbouring Ta-chains. (c), (d) correspond with (a),(b) respectively but with the view direction of [110]. The resulted pattern of (d) mimics the one by TEM observations in Fig.3h in main text. In (a)-(d), the Se atoms are omitted for clear identifications. (e) The perspective view of pristine lattice of Ta_2NiSe_5, the origin rectangles denote the Ta-Ni-Ta sub-units. The red arrow denotes the weakest Ta-Se bonds, which locate between the two nearest-neighbouring Ta-chains.

II. Additional data for coherent phonon spectra

The coherent phonon spectra of the bulk sample were derived from the pump-probe experiments as a comparison to the nano-sheet sample. The fluence dependence of the transient reflectivity spectra and the coherent phonon spectra of the bulk sample were showed in **Supplementary Fig. 6** (a) and (b). They had the same behavior as the nano-sheet sample, showing a disappearance of the phonon mode of 2- and 3.7-THz beyond the threshold. It can also be easily recognized from the spectra of 3 THz with a broadening and a shift to lower frequency with increasing pump power.

However, as shown in **Supplementary Fig. 6** (c) and (d), when we checked the coherent phonon spectra of the bulk sample after the high intense writing pulses, the bulk sample reverted to the pristine state again, indicating no persistent phase transition.

The fluence dependent transient reflectivity spectra for the nano-sheet sample of the pristine Ta_2NiSe_5 are shown in **Supplementary Fig. 7**. The reversible PI phase transition in nano-sheet sample after the moderate intensity pulses is demonstrated in **Supplementary Fig. 8**.

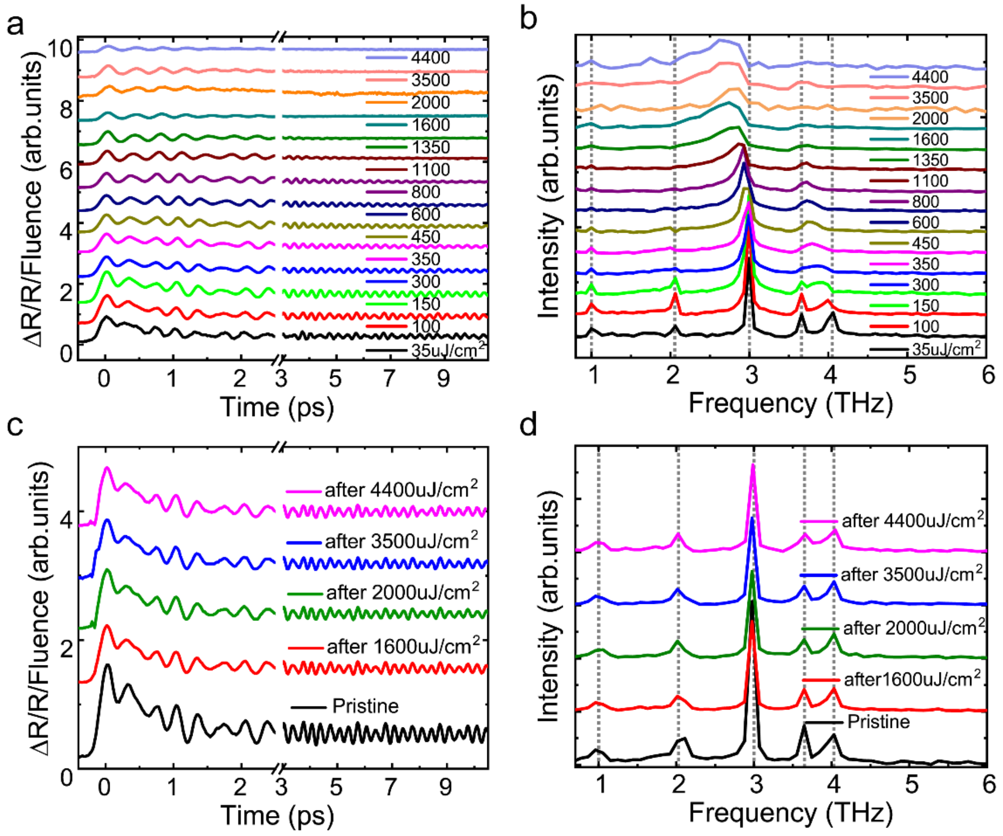

Supplementary Fig. 6. (a) Transient reflectivity spectra. (b) The corresponding amplitude mode spectra of (a) obtained through the fast Fourier Transformation. The data are normalized to pump power. (c) The reflectivity spectra measured after the writing pulse ceased. The fluence of the writing pulse is varied, but the measurements are made with a pump-probe sequence with very low pump and probe power. (d) The corresponding FFTs of (c) from transient reflectivity spectra. There is always a degree of spectral weight from 2.0- and 3.7-THz modes remainder. The sample temperature was 50 K.

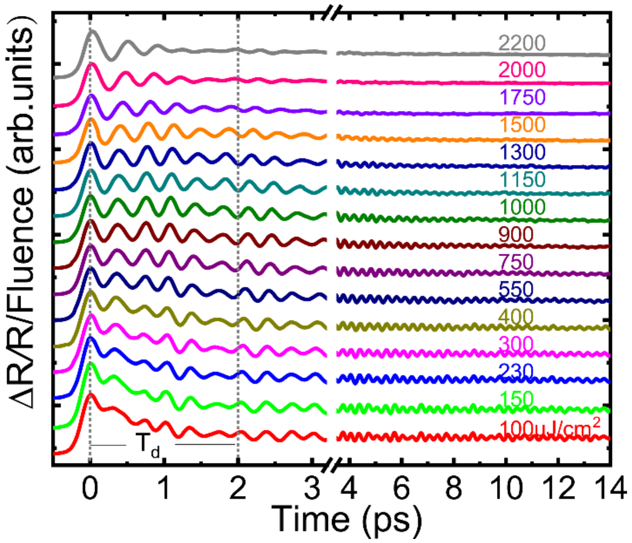

Supplementary Fig. 7. The transient reflectivity *ΔR/R* of the nano-sheet sample at moderate pump pulse fluence. The gray are guidelines for evolution of period of oscillation dependent on the excitation intensity. At high density excitations, the oscillations are retarded. The sample temperature was 50 K.

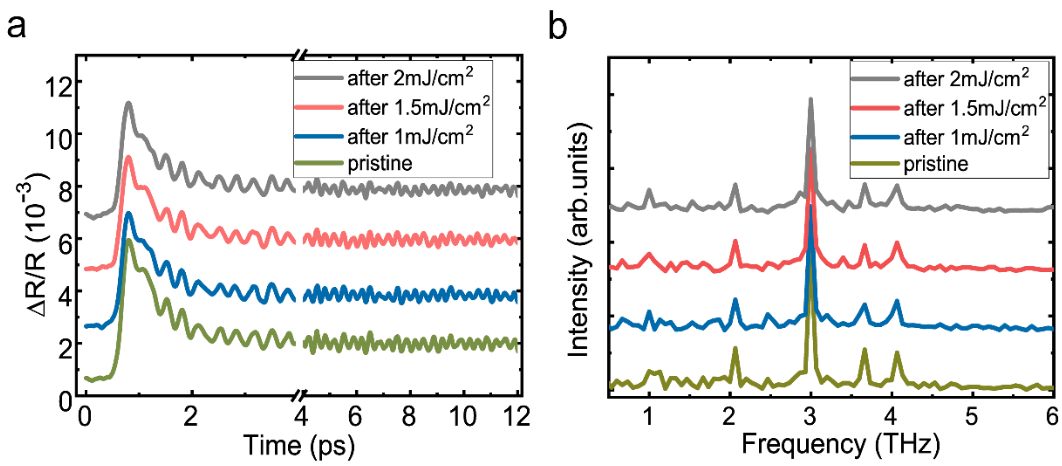

Supplementary Fig. 8. (a) Transient reflectivity spectra measured after the moderate intensity pulse ceased. (b) The corresponding FFTs of (a), showing the 2.0- and 3.7-THz modes recovery. All the data were obtained with 35 fs pulses at 800 nm. The sample temperature was 50 K.

We performed Fourier transformation of the pump-probe waveform after cutting the initial time delay T_d in order to analyze the time-delay evolution of the phonon frequency. The results of the transient phonon spectra for a nano-sheet sample of the pristine Ta_2NiSe_5 at 50 K with excitations up to 2.2 mJ/cm^2 are shown in Fig. S9. At the pump fluence of 1.5 mJ/cm^2, the peak frequency of the nominal '3THz' mode locates roughly at 2.82 THz when the entire pump-probe waveform is used for Fourier transformation. It shifts to ~ 2.98 THz after cutting the initial time delay of 2 ps. After ~ 2 ps, only the mode with a nearly symmetric line shape is observed. Similar situation is seen for other pump fluences, as shown in **Supplementary Fig. 9f**.

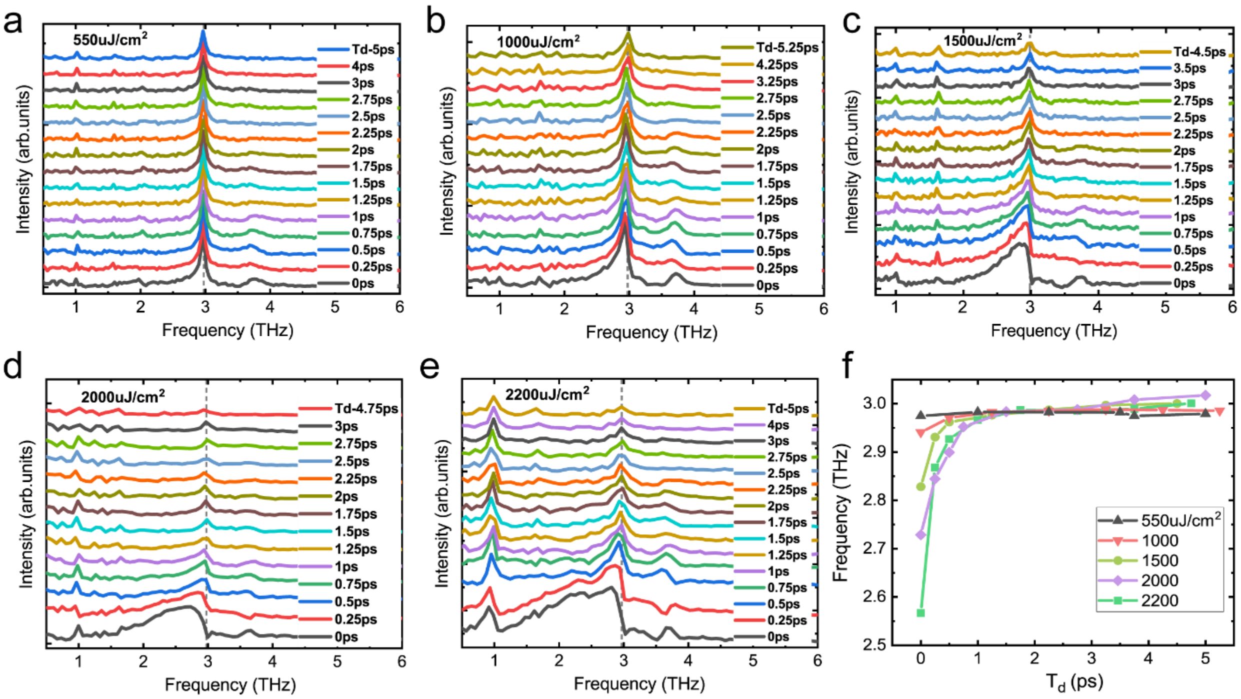

Supplementary Fig. 9. (a-e). Time delay dependence of the transient phonon spectra at various excitation intensity. The anharmonic broadening effect can be easily recognized, especially at high excitations. After ~ 2 ps, only the mode with a nearly symmetric line shape is observed. (f) At high excitations, a clear frequency recovery after a time delay ~ 2 ps can be recognized.

III. Photoinduced low resistance state of Ta_2NiS_5

Ta_2NiS_5 is a layered narrow-gap semiconductor with lattice structure isostructural to Ta_2NiSe_5. The difference is that Ta_2NiS_5 does not demonstrate the structural distortion or anomaly in the resistivity, which are present in Ta_2NiSe_5, and for this reason it is not believed to be an excitonic insulator. We performed similar measurement on Ta_2NiS_5, and found that very similar photoinduced low resistance stable state can also be achieved in Ta_2NiS_5 nano-thickness samples.

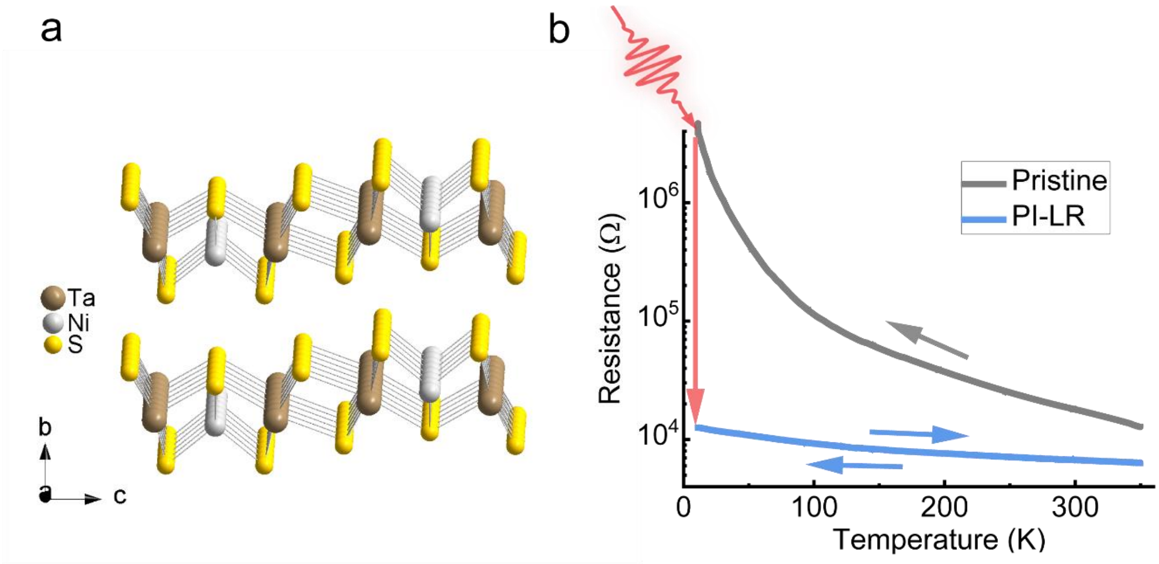

Supplementary Fig. 10 (a) The layered crystal structure of Ta_2NiS_5. (b)The four-probe resistance of the pristine (gray) and PI-LR state (blue) of the Ta_2NiS_5 film at 5K. The writing excitation was ~4.5 mJ/cm^2.

IV. The list of pump laser parameters from different references in the main text.

According to characteristic of the ultrafast laser and the classical electromagnetic theory, the electric field peak intensity of the laser pulses is estimated through the equation:

$$E_{peak} = \sqrt{2Z_{F_0}\frac{F}{\tau_{FWHM}}}$$

where $Z_{F_0} = 377\Omega$, τ_{FWHM} is the pulse duration, $F = \frac{w}{A_{eff}}$ is the pump fluence, w is the single pulse energy, A_{eff} is the area of the beam cross-section.

In Supplementary Table.1, the references number correspond to the ones of the main text.

Supplementary Table. 1. The list of pump laser parameters from different references in the main text.

Group	Centre Wavelength	Pulse Duration	Repetition Frequency	Max. Pump Fluence (mJ /cm^2)	Electric Field Peak (MV/cm)	PI-Phase Transition	Experiment Type
Ref.23	800 nm	110fs	40kHz	0.47	1.8	No	t-ARPES
arXiv:2007.02909	800 nm	230 fs	100 kHz	0.85	1.67	No	t-ARPES
Ref. 47	800 nm	130 fs	/	1.75	3.18	No	fs optical spectra
Ref. 24	800nm	30 fs	1 kHz	1.56	6.26	Yes	t-ARPES
Ref.25	800 nm	35 fs	/	2.27	7.02	Yes	t-ARPES
Ref.48	700 nm	30 fs	500 kHz	0.32	2.83	Yes	t-ARPES
Ref. 49	700 nm	12 fs	/	0.88	7.43	Yes	fs optical spectra
The present work	800 nm	35 fs	1 kHz	3.5	8.68	Yes	fs optical spectra
